# Pervasive Chimerism in the Replication-Associated Proteins of Uncultured Single-Stranded DNA Viruses

**DOI:** 10.3390/v10040187

**Published:** 2018-04-10

**Authors:** Darius Kazlauskas, Arvind Varsani, Mart Krupovic

**Affiliations:** 1 Institute of Biotechnology, Vilnius University, Saulėtekio Av. 7, Vilnius 10257, Lithuania; d.kazlauskas@ibt.lt; 2 Unité Biologie Moléculaire du Gène chez les Extrêmophiles, Department of Microbiology, Institut Pasteur, 25 rue du Docteur Roux, Paris 75015, France; 3 The Biodesign Center for Fundamental and Applied Microbiomics, School of Life Sciences, Center for Evolution and Medicine, Arizona State University, Tempe, AZ 85287, USA; arvind.varsani@asu.edu; 4 Structural Biology Research Unit, Department of Integrative Biomedical Sciences, University of Cape Town, Observatory 7700, South Africa

**Keywords:** CRESS DNA viruses, virus evolution, rolling-circle replication initiation proteins, ssDNA viruses, superfamily 3 helicase domain, HUH endonuclease domain, recombination

## Abstract

Numerous metagenomic studies have uncovered a remarkable diversity of circular replication-associated protein (Rep)-encoding single-stranded (CRESS) DNA viruses, the majority of which are uncultured and unclassified. Unlike capsid proteins, the Reps show significant similarity across different groups of CRESS DNA viruses and have conserved domain organization with the N-terminal nuclease and the C-terminal helicase domain. Consequently, Rep is widely used as a marker for identification, classification and assessment of the diversity of CRESS DNA viruses. However, it has been shown that in certain viruses the Rep nuclease and helicase domains display incongruent evolutionary histories. Here, we systematically evaluated the co-evolutionary patterns of the two Rep domains across classified and unclassified CRESS DNA viruses. Our analysis indicates that the Reps encoded by members of the families *Bacilladnaviridae*, *Circoviridae*, *Geminiviridae*, *Genomoviridae*, *Nanoviridae* and *Smacoviridae* display largely congruent evolutionary patterns in the two domains. By contrast, among the unclassified CRESS DNA viruses, 71% appear to have chimeric Reps. Such massive chimerism suggests that unclassified CRESS DNA viruses represent a dynamic population in which exchange of gene fragments encoding the nuclease and helicase domains is extremely common. Furthermore, purging of the chimeric sequences uncovered six monophyletic Rep groups that may represent new families of CRESS DNA viruses.

## 1. Introduction

The role of genetic recombination in virus evolution cannot be overestimated. It is a dominant force in shaping viral genomes and associated phenotypes, including adaptation, host switching and virus emergence [1,2,3]. Although the rate of recombination varies across virus taxa [4], a considerable body of evidence indicates that it has affected viruses with different genome types and has resulted in gene exchange not only between unrelated viruses, but also between viruses and non-viral mobile genetic elements as well as cellular organisms [5]. The very origin of viruses might be a result of an assortment of genes with different evolutionary histories. For instance, it has been suggested that capsid proteins, a hallmark of viruses, have evolved on multiple independent occasions from refunctionalized cellular proteins [6]. Consequently, the virosphere is an interconnected modular network of gene sharing [7], whereby viral genomes consist of distinct functional modules which are exchanged through recombination between evolutionarily distinct viral lineages. The effects of recombination are particularly pronounced in the case of viruses with small RNA and DNA genomes, because exchange of a single gene produces chimeric entities in which large fractions of the genome have different provenances. 

Viruses with single-stranded (ss) DNA genomes infect hosts in all three domains of life and are among the smallest viruses known [8,9]. These viruses are currently classified by the International Committee on Taxonomy of Viruses (ICTV) into thirteen families (https://talk.ictvonline.org/taxonomy/). Members of the families *Microviridae* and *Inoviridae* infect bacteria, viruses of the families *Spiraviridae* and *Pleolipoviridae* infect archaea, whereas eukaryotes are infected or are associated with viruses that are classified into the families *Anelloviridae*, *Bacilladnaviridae*, *Bidnaviridae*, *Circoviridae*, *Geminiviridae*, *Genomoviridae*, *Nanoviridae*, *Parvoviridae* and *Smacoviridae*. The eukaryotic ssDNA viruses with circular genomes, with the exception of anelloviruses, encode homologous rolling-circle replication-associated protein (Rep) and are unofficially referred to as circular, Rep-encoding single-stranded (CRESS) DNA viruses [9]. The Rep is a multifunctional protein containing an endonuclease and a helicase domain. The N-terminal endonuclease domain is responsible for nicking/joining activity at the origin of DNA replication and contains three conserved motifs (I–III), typical of HUH superfamily endonucleases [8,10]. The C-terminal superfamily 3 helicase domain, responsible for unwinding the dsDNA intermediate, includes four conserved motifs known as Walker A, Walker B, motif C [11] and the ‘Arginine finger’ motif [12]. Numerous metagenomic studies have uncovered an incredible diversity of Reps encoded by CRESS DNA viruses which appear to be widespread in diverse habitats [13,14].

The genomes of CRESS DNA viruses display high substitution rates and are highly recombinogenic [3,15,16,17,18,19,20,21]. A combination of these properties presumably contributes to the rapid diversification and adaptation of these viruses to new environments and hosts. Recent reports based on sequence analysis suggest that viruses with ssDNA genomes can sample genes not only among viruses with DNA but also RNA genomes [12,22,23,24,25]. Furthermore, not only complete genes are exchanged, but recombination also occurs at the level of functional domains. For instance, it has been shown that the nuclease and helicase domains of the Reps in certain CRESS DNA viruses have distinct evolutionary histories [26,27]. However, the extent of such intragenic recombinations across different CRESS DNA virus groups has not been investigated. Notably, genus- and family-level classification of CRESS DNA viruses is often based on the phylogenetic analysis of Reps [28,29]. Given that intragenic recombinants in Rep phylogenies typically occupy a position intermediate between the parental clades [26,27], their inclusion in phylogenetic analyses might blur the evolutionary relationships between different CRESS DNA virus groups. 

Here, we analyzed amino acid sequences of Reps encoded by classified and unclassified eukaryote-associated CRESS DNA viruses and systematically investigated the co-evolutionary patterns of their nuclease and helicase domains. We show that ~71% of Reps encoded by unclassified CRESS DNA viruses are chimeric, with the endonuclease and helicase domains displaying incongruent evolutionary patterns. Removal of the recombinant Rep proteins from the dataset has revealed several coherent groups of uncultivated CRESS DNA viruses, which might represent new virus families.

## 2. Methods

### 2.1. Dataset

Genome sequences of CRESS DNA viruses were downloaded from GenBank (October, 2017). These genomes were sequenced in 137 independent studies by various researchers and are derived from highly diverse samples (Supplementary file 1). The initial dataset contained 647 sequences. One group of Reps encoded by unclassified CRESS DNA viruses was discarded because its helicase motifs were not conserved (Supplementary file 1). After filtering out proteins with differently evolving domains, there were 380 sequences (a 41% reduction). 

### 2.2. Multiple Sequence Alignments and Phylogenetic Analysis

For all sequence alignments, we used MAFFT v7 [30] optimized for accurate local alignment (options “L-INSI-i --leavegappyregion --ep 0.123”). Alignments were trimmed using TrimAl v1.2 [31] with gap threshold of 0.2. Alignments of endonuclease and helicase domains were produced by splitting the full-length Rep sequence alignment used to calculate the tree shown in Figure S1 at the beginning of the conserved Walker A motif. Alignment lengths and number of sequences used are provided in the corresponding figure legends. Sequence alignments used in this study are available from the authors upon request. Phylogenetic trees were generated with PhyML v3.0 using automatic model selection and a fast likelihood-based method (aBayes) for branch support [32].

### 2.3. Detection of Chimeric Rep Proteins

CRESS DNA viruses display considerable sequence diversity. Detection of recombination among diverse viruses is challenging using nucleotide sequence-based recombination detections approaches such as those implemented in specialized tools, such as the widely used RDP (Recombination Detection Program) v4 package [33]. Hence, there is a heavy reliance on analyzing this in protein sequence space with phylogeny-based approaches. For instance, smacoviruses, geminiviruses and genomoviruses display intrafamilial genome-wide sequence diversity of ~45–47% [28,29], whereas application of RDP is not recommended for datasets displaying nucleotide sequence divergence exceeding 30% [33]. Thus, for the highly diverse CRESS DNA viruses, where more ancient recombination events have likely shaped the genomes, analysis of protein sequences is essential. We used two complementary approaches to detect possible recombination in Rep proteins. First, we generated a tanglegram of the Rep endonuclease and helicase trees using Dendroscope v3 [34]. Sequences or sequence groups whose positions differed in both trees were marked as possible recombinants. Second, to substantiate the results, we performed an all-against-all comparison of endonuclease and helicase domain sequences with phmmer (http://hmmer.org). Domain sequences from the tanglegram analysis were used as queries. The top 20% of hits with the lowest e-value across the whole sequence were extracted for each domain pair (nuclease and helicase). The number of common hits (excluding “self” hits) was counted and the resultant number was divided by a total number of hits. Each Rep was assigned a domain “connectedness” score, which represents a probability that the “best” 20% of hits identified via phmmer when utilizing either endonuclease or helicase sequences as queries will be identical. The latter ranges from 0 to 1 (where the probability of <0.2 indicates likely recombination and the number close to 1 indicates that both domains find the same set of proteins). This approach has been tested on the example of CRESS DNA viruses, which were previously found to encode chimeric Reps [26,27] as well as on those that were considered not to be recombinant [26]. The recombinant Reps in this dataset had an average probability of 0.7 (median of 0.67), whereas the chimeric Reps had the average probability of 0.19 (median of 0.19; Supplementary file 1). Thus, the probability of <0.2 indicates likely recombination. 

### 2.4. Sequence Logos

Sequence logos for the Reps of *Geminiviridae*, *Circoviridae* and *Smacoviridae* families were taken from [12]. Alignments for other groups were obtained from an alignment used to build the tree shown in Figure S1. Sequence logos were produced using WebLogo 3 server [35].

## 3. Results and Discussion

### 3.1. Evaluation of Coevolution of the Nuclease and Helicase Domains

**Figure 1:**
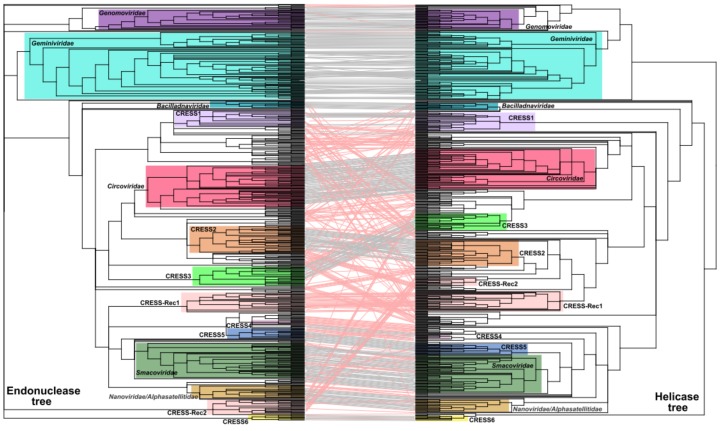
Tanglegram of maximum likelihood phylogenetic trees of the nuclease (left) and helicase (right) domains of Reps encoded by circular, Rep-encoding single-stranded (CRESS) DNA viruses. Putative recombinant proteins are joined with red lines. Clades forming distinct groups are marked with colored rectangles. Branches with support lower than 70% were collapsed. The nuclease and helicase phylogenies were inferred using PhyML [32] with the VT + G (VT matrix; G, gamma shape parameter) and rtREV + G + I + F (rtREV amino acid model; G, gamma shape parameter: fixed; I, proportion of invariable sites: fixed; F, equilibrium frequencies: empirical) substitution models, respectively. The alignments contained 267 and 206 aa positions, respectively. Rec, recombinant Rep group.

To evaluate the congruence between the evolutionary patterns of the nuclease and helicase domains, the corresponding phylogenetic trees were juxtaposed and ordered to maximize the correspondence between the taxa using the binary tanglegram approach (Figure 1). In addition, we developed a scoring-based method to detect the recombination between divergent Rep domains based on the propensity of the two domains to find the same set of viruses among the best hits in all-against-all sequence comparisons (see Methods). The two approaches described above are complementary rather than alternative. The tanglegram approach reveals recombination events that happened in a more distant past, over longer phylogenetic distances, whereas the second approach is more suited for detection of recombination events that involved more closely related sequences (e.g., members of the same family). Due to inherent heterogeneity of the dataset, the two approaches produced somewhat conflicting results. For instance, in the case or CRESS-Rec1 group (see below), the protein domains had high connectedness probability, likely due to ancient recombination event, but displayed different affinities in phylogenetic analyses (Figure 1; Supplementary file 1). Each of such cases was assessed manually. We note that neither of the two methods has sufficient resolution to identify recombinations between closely related genomes. However, detection of such recombination events was outside of the scope of this study where we mainly focus on diverse CRESS DNA virus sequences.

### 3.2. Coherent Evolutionary Patterns in Classified CRESS DNA Viruses 

Analysis of the tanglegram revealed that phylogenies of the nuclease and helicase domains were largely congruent for most of the clades corresponding to established virus families (i.e., *Bacilladnaviridae*, *Circoviridae*, *Geminiviridae*, *Genomoviridae*, *Nanoviridae* and *Smacoviridae*), with only roughly two percent (six out of 286) of Reps showing evidence of distinct evolutionary histories for the two domains. Five of the cases involved animal-associated viruses of the recently created family *Smacoviridae* [29]. In members of the species *Porcine-associated porprismacovirus 2*, *3*, *8* and *9* (genus *Porprismacovirus*), the nuclease and helicase domains show closer similarities to distinct members of the same genus, suggesting an intrageneric recombination. By contrast, in bovine faeces associated smacovirus 4, the sole member of the genus *Cosmacovirus*, the nuclease and helicase domains appear to be derived from smacoviruses classified in genera *Porprismacovirus* and *Drosmacovirus*, respectively. Consistent with this inference, in the full-length Rep phylogeny, bovine faeces associated smacovirus 4 occupies an intermediate position between porprismacoviruses and drosmacoviruses [29]. Similarly, a chimeric Rep is encoded by grapevine red blotch virus, the only representative of the genus *Grablovirus* in the family *Geminiviridae* [36]. The nuclease domain of the latter virus is highly divergent and is not closely related to those of other sequenced CRESS DNA viruses, whereas the helicase domain is most similar to the corresponding domain of alfalfa leaf curl virus, a member of the genus *Capulavirus* (family *Geminiviridae*) [36]. In the full-length Rep phylogenies, grapevine red blotch virus forms a sister group to capulaviruses [36]. The scarcity of cases described above suggests that among classified CRESS DNA viruses recombination events within the rep genes are largely selected against even between relatively closely related viruses (different genera of the same family). Recombination rates can be influenced by various factors, including local degrees of sequence similarity between recombining genomes, DNA secondary structures and genomic sensitivity to nuclease attack or breakage, whereas the viability of recombinant genomes could be influenced by the degree to which their co-evolved genetic interactions are perturbed by recombination [19,20,37,38]. In the case of geminiviruses, it has been experimentally demonstrated that patterns of recombination are strongly influenced by selection against recombinants in which intra-genomic interactions required for proper protein and nucleic acid folding are disrupted [37,39]. Presumably, similar fitness costs prohibit the survival of recombinants within the rep genes of bacilladnaviruses, circoviruses, geminiviruses, genomoviruses, nanoviruses and smacoviruses. However, this tendency appears to be specific to these “firmly” established virus groups, in which the intra-genomic interactions have presumably achieved certain level of optimality in their corresponding habitats. 

### 3.3. High Incidence of Chimerism in the Reps of Unclassified CRESS DNA Viruses 

**Figure 2:**
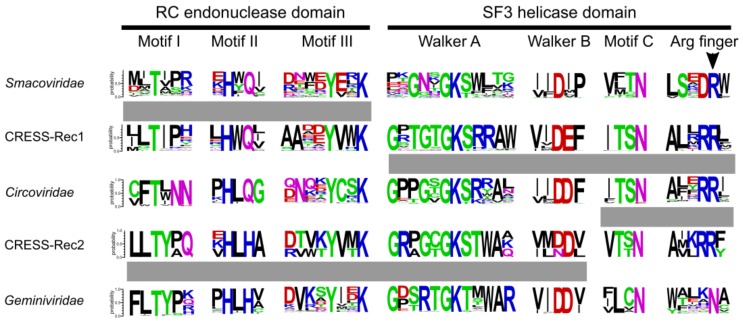
Sequence motifs of Rep proteins from classified and possibly recombinant CRESS DNA viruses. Motifs are presented as sequence logos and those containing predicted recombinations are joined with grey rectangles. Residues are colored according to their chemical properties (polar, green; basic, blue; acidic, red; hydrophobic, black; neutral, purple).

In stark contrast to the classified viruses, ~71% (256 out of 361) of Reps encoded by unclassified CRESS DNA viruses display signs of divergent evolutionary history for the nuclease and helicase domains (Figure 1). Potential recombination was detected across a spectrum of phylogenetic distances, from recombination between members of the same genus to that involving partners belonging to different virus families/clades (Supplementary file 1). The majority of recombinant Rep sequences did not form consistent clades in either nuclease or helicase trees, but were rather scattered among other clades (Figure 1). Interestingly, however, we identified two conserved groups of chimeric Reps, herein referred to as CRESS-Rec1 and CRESS-Rec2, which formed monophyletic groups in both nuclease and helicase trees, but the two displayed incongruent phylogenetic patterns. Members of the CRESS-Rec1 group (*n* = 29) contain the smacovirus-like nuclease domain and a circovirus-like helicase domain (Figure 2), whereas Reps of the CRESS-Rec2 group (*n* = 10) have divergent nuclease domains, distantly related to those of geminiviruses, and helicase domains shared with circoviruses, particularly within motif C and the Arginine finger motif (Figure 2). Viral genomes encoding the Reps from both groups were identified from highly diverse habitats, including animal and environmental samples (Supplementary file 1), suggesting that they are widespread in nature, which, to certain degree, testifies to their evolutionary success. 

The observation that in the evolution of unclassified CRESS DNA viruses the two Rep domains are frequently replaced by homologous domains from distantly related viruses is surprising, especially in the light of scarcity of such exchanges among the classified viruses. Conceivably, under certain circumstances, for instance, in the cases of population bottlenecks that could occur during inter-host transmission in the environment, genome repair by recombination may be the only solution to avoid extinction, even if the fitness of the recombinant virus is relatively low due to disruption of co-evolved genetic interactions. In the case of geminiviruses, it has been shown that co-inoculation of two severely defective viruses yields recombinant progeny which were far more fit than their parents [38]. Notably, recombinant Reps of unclassified viruses display similarities to particular domains from all families of classified CRESS DNA viruses, except for bacilladnaviruses. This suggests that viruses from the recognized virus families contribute to the global gene pool which is sampled by diverse CRESS DNA viruses.

### 3.4. Six Potentially New Families of CRESS DNA Viruses 

**Figure 3:**
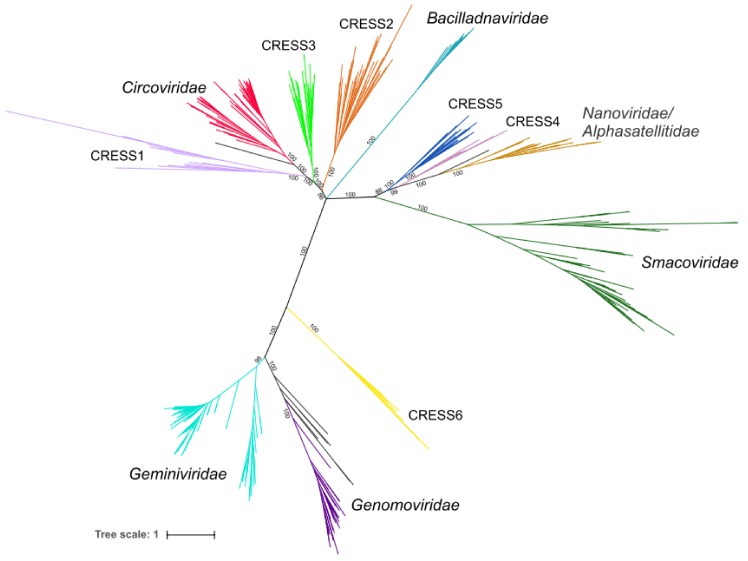
Phylogenetic tree of CRESS viruses generated after removing possible recombinant proteins. The tree is based on full-length Rep amino acid sequences. Clades belonging to the same group have the same colors as in Figure 1. Edges having support lower than 70% were contracted. The phylogeny was inferred using PhyML [32] with the rtREV+G+F (rtREV amino acid model; G, gamma shape parameter: fixed; F, equilibrium frequencies: empirical) substitution model using the alignment containing 412 positions.

Chimeric proteins might distort the conclusions drawn from phylogenetic analyses due to conflicting similarities of distinct protein domains. Indeed, maximum likelihood phylogenetic analysis of the 647 full-length Rep sequences present in our dataset produced a star-shaped phylogeny, with poorly resolved basal branches (Figure S1). Thus, to gain better understanding on the global relationships between the major groups of CRESS DNA viruses, we removed from our dataset sequences of all Reps in which nuclease and helicase domains showed incongruent evolutionary patterns and repeated the phylogenetic analysis. In the resultant phylogenetic tree (Figure 3), all previously established families of CRESS DNA viruses are recovered as monophyletic with maximal statistical support (except for the *Geminiviridae* clade which has a support of 98%). Our analysis also revealed six groups of unclassified CRESS DNA virus groups in which most of the members displayed congruent evolutionary patterns for the nuclease and helicase domains. These groups were tentatively labeled CRESS1 through 6 (Figure 1; Supplementary file 1). In both nuclease and helicase trees, members of CRESS1, 2 and 3 branched with circoviruses, whereas CRESS4 and 5 domains showed stronger affinity to the corresponding domains of nanoviruses and smacoviruses (Figure 1). By contrast, members of the CRESS6 group were more divergent and generally branched separately from the other virus groups.

In the phylogeny constructed from the full-length Reps, all six virus groups were also monophyletic, with maximum support (Figure 3). CRESS1–3 occupied a basal position to members of the family *Circoviridae*, whereas CRESS4 and CRESS5 are at the base of the clade including nanoviruses and alphasatellites (family *Alphasatellitidae*) [40]. CRESS6 group, as in single domain phylogenies (Figure 1), was not closely related to other virus groups, but showed affinity to the clade including *Geminiviridae* and *Genomoviridae* (Figure 3). The viruses encoding Reps from each of the six groups appear to be widely distributed in nature, because their genomes were recovered from samples collected from diverse sources, including various vertebrates, arthropods, marine environments, sewage, etc. (Supplementary file 1). Notably, Rep sequences within the six groups display divergence comparable to that among viruses within recognized families. It is thus possible, if not likely, that CRESS1–6 groups represent new families of CRESS DNA viruses. However, further studies, such as analysis of the corresponding capsid proteins, are needed to validate this assertion and will be described elsewhere. 

## 4. Conclusions

Various studies have recently shown that CRESS DNA viruses are a major, highly diverse component of the global virome and, in certain environments, represent the dominant virus group [9,13,41,42,43,44,45,46,47,48,49,50,51,52]. The majority of these viruses has been discovered through metagenomics approaches and are uncultured. Thus, our understanding on the diversity and impact of CRESS DNA viruses on their hosts and the environment are still scarce. Here, by analyzing the evolutionary patterns of the key replication protein, Rep, shared by all CRESS DNA viruses, we show that the Reps of these viruses are dynamic and that recombination within the Rep is highly prevalent, with ~71% of unclassified CRESS DNA viruses encoding chimeric Reps. Nevertheless, such recombination events are rare among viruses from established families, suggesting that pairing of the two domains in these virus groups is optimized for particular hosts/environments and interfamilial recombinations are largely unfavorable. It remains to be determined whether the rep genes are hotspots for recombination in uncultivated CRESS DNA viruses or if recombination within the intergenic regions resulting in the exchange of the Rep- and capsid-encoding genes occurs with the same or perhaps even higher frequency. Indeed, previous studies have shown that shuffling of the two major viral genes occurs both among members of the same families [28,29] as well as between evolutionarily unrelated virus lineages [8,23,25]. Our analysis has uncovered six groups of potential new CRESS DNA virus families. It will be interesting to explore the extent of capsid protein diversity associated with these new virus groups. Notably, the six virus groups occupy basal positions to the major groups of cultivated CRESS DNA viruses, including *Circoviridae* (CRESS1–3), *Nanoviridae* (CRESS4 and CRESS5) and *Geminiviridae* (CRESS6). Detailed analysis of these virus groups may provide valuable insights into the origin and evolution of these “classical” groups of CRESS DNA viruses, many of which infect cultivated crops or livestock and are of significant economic importance. More generally, our results reinforce the notion that modularity, whereby functional domains with different evolutionary histories are assorted by recombination to produce novel genetic variants, is a pervasive theme across the virosphere.

## Supplementary Materials

The following are available online at http://www.mdpi.com/1999-4915/10/4/187/s1, Supplementary file 1: List of Rep proteins analyzed in this study, Figure S1: Phylogenetic tree of all CRESS DNA viruses based on full-length Rep amino acid sequences.

## References

[1] Dennehy J.J. (2017). Evolutionary ecology of virus emergence. Ann. N. Y. Acad. Sci..

[2] Holmes E.C., Drummond A.J. (2007). The evolutionary genetics of viral emergence. Curr. Top. Microbiol. Immunol..

[3] Lefeuvre P., Moriones E. (2015). Recombination as a motor of host switches and virus emergence: Geminiviruses as case studies. Curr. Opin. Virol..

[4] Simon-Loriere E., Holmes E.C. (2011). Why do RNA viruses recombine?. Nat. Rev. Microbiol..

[5] Koonin E.V., Dolja V.V., Krupovic M. (2015). Origins and evolution of viruses of eukaryotes: The ultimate modularity. Virology.

[6] Krupovic M., Koonin E.V. (2017). Multiple origins of viral capsid proteins from cellular ancestors. Proc. Natl. Acad. Sci. USA.

[7] Iranzo J., Krupovic M., Koonin E.V. (2016). The double-stranded DNA virosphere as a modular hierarchical network of gene sharing. mBio.

[8] Krupovic M. (2013). Networks of evolutionary interactions underlying the polyphyletic origin of ssDNA viruses. Curr. Opin. Virol..

[9] Rosario K., Duffy S., Breitbart M. (2012). A field guide to eukaryotic circular single-stranded DNA viruses: Insights gained from metagenomics. Arch. Virol..

[10] Chandler M., de la Cruz F., Dyda F., Hickman A.B., Moncalian G., Ton-Hoang B. (2013). Breaking and joining single-stranded DNA: The HUH endonuclease superfamily. Nat. Rev. Microbiol..

[11] Gorbalenya A.E., Koonin E.V., Wolf Y.I. (1990). A new superfamily of putative NTP-binding domains encoded by genomes of small DNA and RNA viruses. FEBS Lett..

[12] Kazlauskas D., Dayaram A., Kraberger S., Goldstien S., Varsani A., Krupovic M. (2017). Evolutionary history of ssDNA bacilladnaviruses features horizontal acquisition of the capsid gene from ssRNA nodaviruses. Virology.

[13] Rosario K., Breitbart M. (2011). Exploring the viral world through metagenomics. Curr. Opin. Virol..

[14] Simmonds P., Adams M.J., Benko M., Breitbart M., Brister J.R., Carstens E.B., Davison A.J., Delwart E., Gorbalenya A.E., Harrach B. (2017). Consensus statement: Virus taxonomy in the age of metagenomics. Nat. Rev. Microbiol..

[15] Duffy S., Holmes E.C. (2009). Validation of high rates of nucleotide substitution in geminiviruses: Phylogenetic evidence from East African cassava mosaic viruses. J. Gen. Virol..

[16] Firth C., Charleston M.A., Duffy S., Shapiro B., Holmes E.C. (2009). Insights into the evolutionary history of an emerging livestock pathogen: Porcine circovirus 2. J. Virol..

[17] Grigoras I., Timchenko T., Grande-Perez A., Katul L., Vetten H.J., Gronenborn B. (2010). High variability and rapid evolution of a nanovirus. J. Virol..

[18] Harkins G.W., Delport W., Duffy S., Wood N., Monjane A.L., Owor B.E., Donaldson L., Saumtally S., Triton G., Briddon R.W. (2009). Experimental evidence indicating that mastreviruses probably did not co-diverge with their hosts. Virol. J..

[19] Lefeuvre P., Lett J.M., Varsani A., Martin D.P. (2009). Widely conserved recombination patterns among single-stranded DNA viruses. J. Virol..

[20] Martin D.P., Biagini P., Lefeuvre P., Golden M., Roumagnac P., Varsani A. (2011). Recombination in eukaryotic single stranded DNA viruses. Viruses.

[21] Padidam M., Sawyer S., Fauquet C.M. (1999). Possible emergence of new geminiviruses by frequent recombination. Virology.

[22] Krupovic M., Koonin E.V. (2014). Evolution of eukaryotic single-stranded DNA viruses of the *Bidnaviridae* family from genes of four other groups of widely different viruses. Sci. Rep..

[23] Stedman K.M. (2015). Deep recombination: RNA and ssDNA virus genes in DNA virus and host genomes. Annu. Rev. Virol..

[24] Diemer G.S., Stedman K.M. (2012). A novel virus genome discovered in an extreme environment suggests recombination between unrelated groups of RNA and DNA viruses. Biol. Direct.

[25] Roux S., Enault F., Bronner G., Vaulot D., Forterre P., Krupovic M. (2013). Chimeric viruses blur the borders between the major groups of eukaryotic single-stranded DNA viruses. Nat. Commun..

[26] Krupovic M., Zhi N., Li J., Hu G., Koonin E.V., Wong S., Shevchenko S., Zhao K., Young N.S. (2015). Multiple layers of chimerism in a single-stranded DNA virus discovered by deep sequencing. Genome Biol. Evol..

[27] Quaiser A., Krupovic M., Dufresne A., Francez A.J., Roux S. (2016). Diversity and comparative genomics of chimeric viruses in Sphagnum-dominated peatlands. Virus Evol..

[28] Varsani A., Krupovic M. (2017). Sequence-based taxonomic framework for the classification of uncultured single-stranded DNA viruses of the family *Genomoviridae*. Virus Evol..

[29] Varsani A., Krupovic M. (2018). *Smacoviridae*: A new family of animal-associated single-stranded DNA viruses. Arch. Virol..

[30] Katoh K., Standley D.M. (2013). MAFFT multiple sequence alignment software version 7: Improvements in performance and usability. Mol. Biol. Evol..

[31] Capella-Gutierrez S., Silla-Martinez J.M., Gabaldon T. (2009). trimAl: A tool for automated alignment trimming in large-scale phylogenetic analyses. Bioinformatics.

[32] Lefort V., Longueville J.E., Gascuel O. (2017). SMS: Smart Model Selection in PhyML. Mol. Biol. Evol..

[33] Martin D.P., Murrell B., Golden M., Khoosal A., Muhire B. (2015). RDP4: Detection and analysis of recombination patterns in virus genomes. Virus Evol..

[34] Huson D.H., Scornavacca C. (2012). Dendroscope 3: An interactive tool for rooted phylogenetic trees and networks. Syst. Biol..

[35] Crooks G.E., Hon G., Chandonia J.M., Brenner S.E. (2004). WebLogo: A sequence logo generator. Genome Res..

[36] Varsani A., Roumagnac P., Fuchs M., Navas-Castillo J., Moriones E., Idris A., Briddon R.W., Rivera-Bustamante R., Murilo Zerbini F., Martin D.P. (2017). *Capulavirus* and *Grablovirus*: Two new genera in the family *Geminiviridae*. Arch. Virol..

[37] Martin D.P., Lefeuvre P., Varsani A., Hoareau M., Semegni J.Y., Dijoux B., Vincent C., Reynaud B., Lett J.M. (2011). Complex recombination patterns arising during geminivirus coinfections preserve and demarcate biologically important intra-genome interaction networks. PLoS Pathog..

[38] Monjane A.L., Martin D.P., Lakay F., Muhire B.M., Pande D., Varsani A., Harkins G., Shepherd D.N., Rybicki E.P. (2014). Extensive recombination-induced disruption of genetic interactions is highly deleterious but can be partially reversed by small numbers of secondary recombination events. J. Virol..

[39] Muhire B.M., Golden M., Murrell B., Lefeuvre P., Lett J.M., Gray A., Poon A.Y., Ngandu N.K., Semegni Y., Tanov E.P. (2014). Evidence of pervasive biologically functional secondary structures within the genomes of eukaryotic single-stranded DNA viruses. J. Virol..

[40] Briddon R.W., Martin D.P., Roumagnac P., Navas-Castillo J., Fiallo-Olivé E., Moriones E., Lett J.-M., Zerbini F.M., Varsani A. (2018). *Alphasatellitidae:* A new family with two subfamilies for the classification of geminivirus- and nanovirus-associated alphasatellites. Arch. Virol..

[41] Yoshida M., Mochizuki T., Urayama S.I., Yoshida-Takashima Y., Nishi S., Hirai M., Nomaki H., Takaki Y., Nunoura T., Takai K. (2018). Quantitative Viral Community DNA Analysis Reveals the Dominance of Single-Stranded DNA Viruses in Offshore Upper Bathyal Sediment from Tohoku, Japan. Front. Microbiol..

[42] Dayaram A., Goldstien S., Arguello-Astorga G.R., Zawar-Reza P., Gomez C., Harding J.S., Varsani A. (2015). Diverse small circular DNA viruses circulating amongst estuarine molluscs. Infect. Genet. Evol..

[43] Cheung A.K., Ng T.F., Lager K.M., Alt D.P., Delwart E., Pogranichniy R.M. (2015). Identification of several clades of novel single-stranded circular DNA viruses with conserved stem-loop structures in pig feces. Arch. Virol..

[44] Ng T.F., Zhang W., Sachsenroder J., Kondov N.O., da Costa A.C., Vega E., Holtz L.R., Wu G., Wang D., Stine C.O. (2015). A diverse group of small circular ssDNA viral genomes in human and non-human primate stools. Virus Evol..

[45] Phan T.G., da Costa A.C., Del Valle Mendoza J., Bucardo-Rivera F., Nordgren J., O’Ryan M., Deng X., Delwart E. (2016). The fecal virome of South and Central American children with diarrhea includes small circular DNA viral genomes of unknown origin. Arch. Virol..

[46] Wang H., Li S., Mahmood A., Yang S., Wang X., Shen Q., Shan T., Deng X., Li J., Hua X. (2018). Plasma virome of cattle from forest region revealed diverse small circular ssDNA viral genomes. Virol. J..

[47] Rosario K., Dayaram A., Marinov M., Ware J., Kraberger S., Stainton D., Breitbart M., Varsani A. (2012). Diverse circular ssDNA viruses discovered in dragonflies (*Odonata*: *Epiprocta*). J. Gen. Virol..

[48] Rosario K., Duffy S., Breitbart M. (2009). Diverse circovirus-like genome architectures revealed by environmental metagenomics. J. Gen. Virol..

[49] Rosario K., Schenck R.O., Harbeitner R.C., Lawler S.N., Breitbart M. (2015). Novel circular single-stranded DNA viruses identified in marine invertebrates reveal high sequence diversity and consistent predicted intrinsic disorder patterns within putative structural proteins. Front. Microbiol..

[50] Dayaram A., Galatowitsch M.L., Arguello-Astorga G.R., van Bysterveldt K., Kraberger S., Stainton D., Harding J.S., Roumagnac P., Martin D.P., Lefeuvre P. (2016). Diverse circular replication-associated protein encoding viruses circulating in invertebrates within a lake ecosystem. Infect. Genet. Evol..

[51] Blinkova O., Victoria J., Li Y., Keele B.F., Sanz C., Ndjango J.B., Peeters M., Travis D., Lonsdorf E.V., Wilson M.L. (2010). Novel circular DNA viruses in stool samples of wild-living chimpanzees. J. Gen. Virol..

[52] Krupovic M., Ghabrial S.A., Jiang D., Varsani A. (2016). *Genomoviridae*: A new family of widespread single-stranded DNA viruses. Arch. Virol..

