# Supplementary material for: Pervasive Chimerism in the Replication-Associated Proteins of Uncultured Single-Stranded DNA Viruses
Source: Viruses. 2018 Apr 10;10(4):187. doi: 10.3390/v10040187 (PMC5923481; doi:10.3390/v10040187)
Supplement: Supplementary file 1 [file viruses-10-00187-s001.zip › Figure S1.pdf]

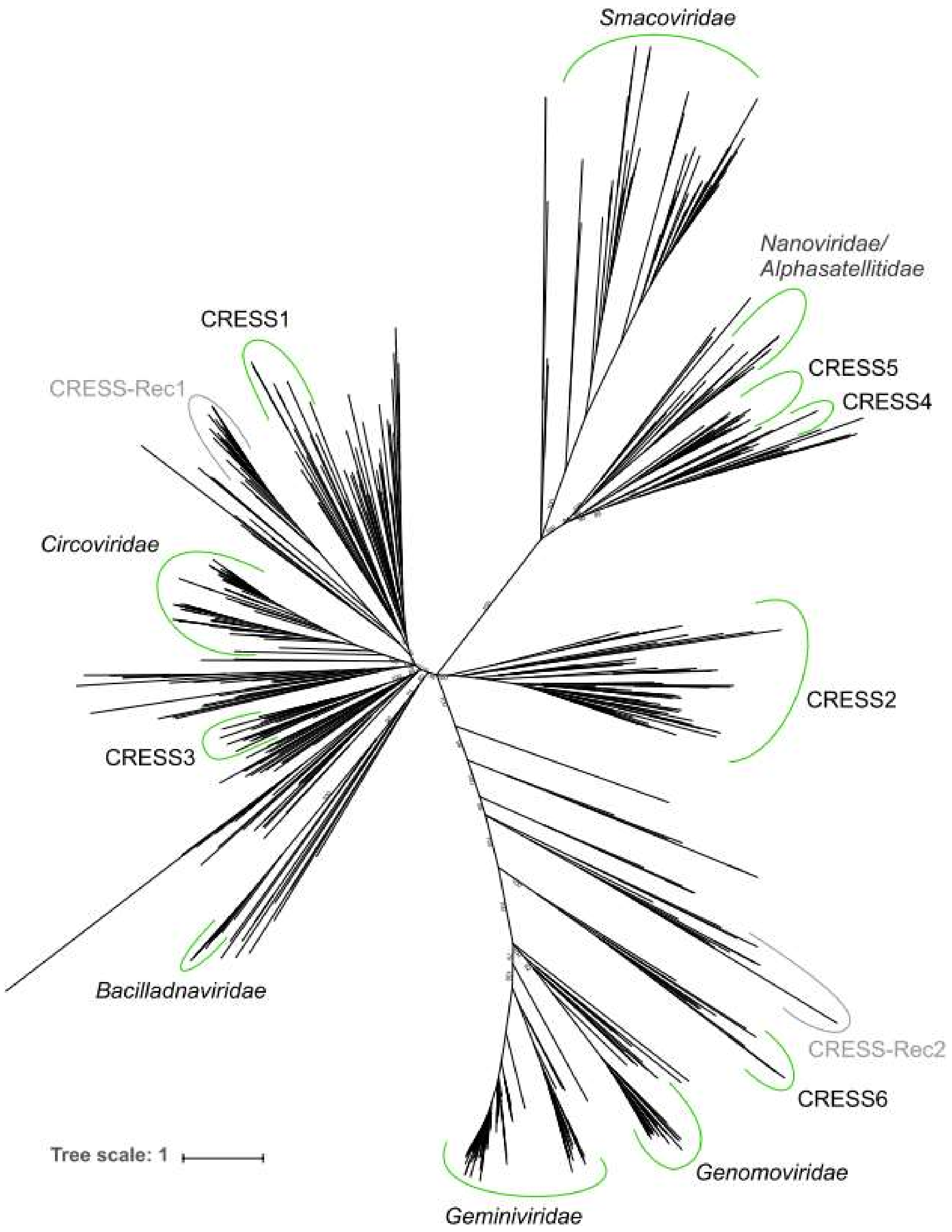

Figure S1. Phylogenetic tree of all CRESS DNA viruses based on full-length Rep amino acid sequences. Clades containing classified CRESS DNA virus groups and those identified in this study are marked. Branches with support lower than 70% were collapsed. The phylogeny was inferred using PhyML [32] with the rtREV+G+F substitution model using the alignment containing 473 positions.
